# Low confidence in the cumulative evidence for the existence of a volume–outcome relationship after revision total knee replacement: A systematic review and meta‐analysis

**DOI:** 10.1002/ksa.12641

**Published:** 2025-03-11

**Authors:** Alexander H. Matthews, Thomas Stringfellow, Hayley Redman, William K. Gray, Jonathan P. Evans, Jonathan T. Evans, Sarah E. Lamb, Tim Briggs, Andrew Price, Andrew D. Toms

**Affiliations:** ^1^ Getting It Right First Time Programme, NHS England London UK; ^2^ Royal Devon University Healthcare NHS Foundation Trust Exeter UK; ^3^ University of Exeter Exeter UK; ^4^ Nuffield Department of Orthopaedics, Rheumatology, and Musculoskeletal Sciences University of Oxford Oxford UK; ^5^ Royal National Orthopaedic Hospital London UK; ^6^ Nuffield Orthopaedic Centre Oxford UK

**Keywords:** complications, hospital volume, re‐revision, revision total knee replacement, surgeon volume

## Abstract

**Purpose:**

This systematic review and meta‐analysis aimed to establish the relationship between the number of procedures a hospital or surgeon performs with outcomes following revision knee replacement (RevKR).

**Methods:**

MEDLINE and Embase were searched using Ovid silver platter up to December 2024 for randomised controlled trials and cohort studies that reported RevKR volumes, in at least two categories, performed by hospitals and surgeons and their relationship to patient and provider level outcomes. The primary outcome was re‐revision rate. Secondary outcomes included mortality, post‐operative complications, patient‐reported outcomes measures (PROMs), emergency readmissions and hospital length of stay. The effect estimates were pooled and plotted using a random‐effects, non‐linear dose–response meta‐analysis (DRMA). Where limitations in the data prohibited DRMA, a narrative approach was utilised. ROBINS‐I and the GRADE approach were used to assess the risk of bias and the confidence in the cumulative evidence, respectively.

**Results:**

A total of 10 cohort studies with data from 1993 to 2021 were included. The confidence in the cumulative evidence exploring the relationship between surgeon/hospital volume and all outcomes after RevKR was very low. An inconsistent relationship was seen between hospital and surgeon volume and re‐revision at any point. There was a non‐linear dose–response relationship between higher hospital volume and lower odds of adverse post‐operative events (*p* < 0.05, *n* = 3 studies, *n* = 35,524 patients). There was no association between increased surgeon volumes and improvements in PROMs (*n* = 2 studies, *n* = 2289).

**Conclusion:**

There is a lack of high‐quality studies establishing the relationship between the number of procedures a hospital or surgeon performs and outcomes following RevKR. Studies are limited to observational designs and are difficult to effectively power due to the rarity of outcomes. Pooling data from multiple studies provides valuable insights but highlights significant heterogeneity and limitations in the existing literature.

**Level of Evidence:**

Level III, systematic review—lowest level of evidence analysed—was from retrospective cohort study of prospectively collected data.

**Abbreviations**
CI: confidence interval
GRADE: Grading of Recommendations, Assessment, Development and Evaluations
OR: odds ratio
PRISMA: Preferred Reporting Items for Systematic Reviews and Meta‐Analyses
PROMIS‐PF 10a: Patient‐Reported Outcomes Measurement Information System Physical Function Short Form 10a
PROM: patient‐reported outcome measure
PROSPERO: international prospective register of systematic reviews
RevKR: revision knee replacement
SWiM: Systematic review Without Meta‐analysis
UK: United Kingdom

## INTRODUCTION

Several studies have shown higher surgical volume is associated with fewer adverse patient outcomes following primary hip and knee replacement surgery [19, 34]. The most recent systematic review exploring this relationship in revision total knee replacement (RevKR) was published in 2012 [8]. This study concluded that increased hospital and surgeon volume decreases adverse patient outcomes [8]. However, at the time, there was limited evidence in the literature and comparing outcomes was complicated by the heterogeneity of the selected study population, hospital volume categories and follow‐up duration. The studies were also dominated by literature from the United States, where differences in healthcare delivery models may introduce bias. Financial incentives for high‐volume centres could influence case‐mix selection, potentially overestimating the impact of higher surgical volumes.

More recently, an emerging body of evidence from Europe has been published, which focuses on the incidence of re‐revision surgery as the most relevant marker of failure [14, 38, 39]. The potential non‐linear relationship between surgical volume and re‐revision surgery was also recently highlighted [39]. Previous evidence in support of the existence of a volume–outcome relationship has been used to redesign the provision of RevKR services in England [5]. The setting of minimum volume targets will call for more revisions to be shifted to specialist centres [15]. The redistribution of workload may negatively affect patient's access to care by creating logistical and financial challenges. It may also potentially increase waiting times for revision surgery. Therefore, it is important to ensure any changes in practice are based on recent evidence which cumulatively supports the existence of a volume–outcome relationship.

A systematic review of the most up‐to‐date literature was undertaken, and a dose‐response meta‐analysis was conducted with the objective of assessing whether surgeon or hospital volume influences patient outcomes in RevKR. Patient‐relevant outcomes for evaluating success have focussed on survivorship of the implants, complications following surgery, patient‐reported outcome measures (PROMs) and hospital impact measures [31]. The null hypothesis was that surgical volume was not associated with any patient‐relevant outcome. The alternative hypotheses were that higher surgical volume was associated with a lower re‐revision rate, a lower mortality rate, a lower rate of any serious medical complications, improved knee function and quality of life, reduced number of emergency readmissions and reduced length of stay.

## METHODS

A systematic review and meta‐analysis were conducted following a pre‐defined protocol registered with PROSPERO (CRD 42025635476) and complying with PRISMA guidelines. This protocol was based on a previously published example investigating the volume–outcome relationship association with primary TKR [18]. A search strategy using keywords and MeSH terms relating to revision knee replacement (RevKR) and surgical caseload was used in the databases MEDLINE and EMBASE accessed through OVID Silver Platter. Please see Supporting Information S1 for a full list of search terms. The databases were searched from their commencement to 31 December 2024. The strategy development was guided by previously published search strategies exploring volume–outcome relationships in arthroplasty [26]. Further sources of literature searched were reference lists of included studies and forward citation searching using the Web of Science.

### Article screening

Studies were included if they (1) involved patients undergoing RevKR, (2) reported data for at least two different hospital volumes or surgeon volumes and (3) analysed at least one of the primary or secondary outcomes. Primary outcomes were re‐revision rate reported at any time point. Secondary outcomes included mortality for up to 1 year, presence of post‐operative complications for up to 1 year; any PROMs, emergency readmissions, surgeon operating times and hospital length of stays. Conference abstracts and review articles were excluded. After duplicates were removed using automated tools, two reviewers (AHM and HR) independently screened the titles and abstracts of all retrieved sources in the web application Rayyan [24]. Arbitration of conflict was performed following consultation with a third reviewer (TS).

### Data extraction

Data were extracted independently by two reviewers using standardised data extraction sheets. Any discrepancies were resolved by consensus. The data items included patient, hospital, and surgeon characteristics; time and country of data collection; data source; surgical volume definitions; RevKR details; primary and secondary outcomes; and statistical analysis details (effect size types, confidence intervals (CIs) and confounding factors). The primary outcome was re‐revision following index RevKR. The secondary outcomes were mortality, adverse post‐operative events, PROMs, hospital length of stay, emergency readmissions, surgeon operating times and cost. Study results (adjusted and/or unadjusted) were extracted separately for each hospital volume category and outcome. For inclusion, studies reporting PROMs following RevKRs required a minimum of 6‐month follow‐up. Both functional and quality of life PROMs were collected.

### Risk of bias

The risk of bias in the included studies was independently assessed at the outcome level by two reviewers using the Risk Of Bias In Non‐randomised Studies of Interventions (ROBINS‐I) tool [36].

### Statistical analysis

Surgical volume was defined as the annual number of patients undergoing RevKR per hospital or surgeon. This was standardised to ensure studies have comparable volume groups. Where volume was recorded in a different format, a weighted estimate was calculated for 1‐year volume. Surgical volume categories were then standardised using their midpoints following a previously published methodology to enable subsequent analysis of cumulative evidence [26]. For individual study outcomes, odds ratios (ORs) with 95% CIs were standardised by setting the lowest hospital volume category as the reference group, following the recommended methodology [37]. For missing ORs, we estimated these using event data following the Chene and Thompson method [7], with CIs calculated using a logarithmic transformation of the ORs. In cases where the OR was equal to 1, indicating no difference, the volume of the reference group was assigned a value of 0. Where different effect measures were used between studies (e.g., hazard ratios [HRs] and ORs), direct conversion of HRs to ORs was not performed due to high levels of heterogeneity between studies [25]. The log OR for each study's results were plotted to visually inspect for non‐linearity for each outcome [35]. A two‐stage random‐effects dose–response meta‐analysis (DRMA) according to Greenland and Longnecker [12] was used to pool ORs for outcomes reported in at least three studies with sufficient data. Both linear and non‐linear models were tested, and the model with the lowest deviance was chosen [2]. Where the non‐linear model was chosen, quadratic models were preferred over restricted cubic splines due to lower model deviance and easier interpretability [35]. Studies with only one non‐reference dose group could not be modelled. All meta‐analyses were performed with R version 3 (R Foundation for Statistical Computing, Vienna, Austria) using the metafor and dosresmeta packages. Outcomes reported in fewer than three studies were not synthesised using meta‐analysis to avoid biased pooled estimates. In such cases, a narrative synthesis using vote counting based on the direction of effect was conducted following the Synthesis Without Meta‐analysis (SWiM) approach [6]. Vote counting applied more weight to larger studies contributing a greater proportion of the data in the formation of overall conclusions.

### Grading the evidence

Confidence in the cumulative evidence was evaluated using the Grading of Recommendations, Assessment, Development and Evaluation (GRADE) approach [26] and applying Murad's approach for SWiM outcomes [23]. This assessment was performed by two reviewers (AHM and TS) independently using the GRADEpro GDT software. A summary of findings tables was prepared for the seven most important outcomes agreed between reviewers. For all domains of GRADE, a weighted assessment of the studies was carried out with reference to the study population and number of events. If the overall risk of bias for each outcome varied between studies, the proportion of patients involved in each study would contribute to the final bias grading [13]. Only the outcomes of critical importance are fully discussed in the results.

## RESULTS

### Study identification and selection

**Figure 1:**
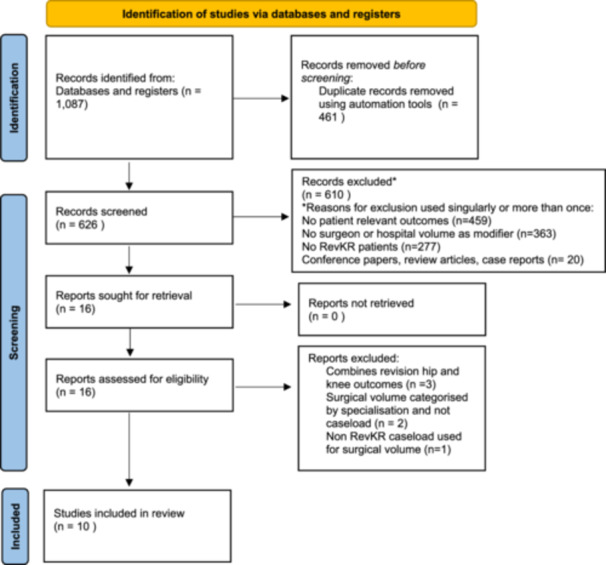
Prisma flow diagram for new systematic reviews, which included searches of databases and registers only.

A total of 1087 records were identified from electronic databases and registers. Of 626 titles and abstracts screened, 610 were excluded. Of the 16 full‐text reports, a further 6 were excluded for reasons shown in Figure 1. This review included 10 cohort studies reporting on the association between hospital volume and/or surgeon volume, with data representing the years from 1993 to 2021.

### Study and patient characteristics

Most studies used data from Europe and North America, while one study obtained data from New Zealand. Please refer to Table 1. The data were obtained from administrative databases in four studies, clinical registries in five studies and regional hospital databases in two studies. The median number of patients across all studies was 6484, with a median of 55% females (range 51%–66%, data from nine studies). Seven studies reported outcomes for hospital annual volume compared with only four studies reporting for surgeon volume. Across the studies, there was a median of 63 hospitals (range: 2–956 in seven studies) and a median of 16 surgeons (range: 8–24 in two studies). There was substantial heterogeneity between the indication for revision in the study populations, with four studies reporting on results in an entirely non‐infected RevKR population, two studies reporting results in a septic cohort of patients and the remaining studies on both septic and aseptic populations.

**Table 1:** Study characteristics and volume/caseload calculations.

Study	Study characteristics								Patients' characteristics					Volume categories (per year)			
Study (ref.)	Type of funding	Country (region)	Primary data source(Registry/Database)	Data coll. (years)	No. of hospitals	No. of surgeons	No. of revisions	No. of patients	Indication for revision	Adjusted for previous revisions	Adjusted for component type revised	% Female	Age (years)	Type	Hospital or surgeon	Annual Volume calculation	Categories
Halder et al. 2020 [4]	No funding	Germany	Administrative	2013–2017	956	NA	23,644	21,573	Aseptic	Yes	Yes	66%	70 (IQR 61–77)	Categorical	Hospital only	Yes	<13/year, 13–24, 25–52, >52
Feinglass et al. 2004 [9]	No funding	USA	Administrative	1993–1999	63	ns	2986	2986	All	No	No	64%	Mean 69 years	Categorical	Hospital only	Yes	<7/year, 7–14/year, >14/year
Samuel et al. 2020 [10]	No funding	USA	Administrative	2007–2012	ns	ns	NA	13,751	Septic group	No	No	51%	Categorised	Centiles	Hospital only	No	<25th percentile, 25–75th percentile, >75th percentile
Van Rensch et al. 2023 [6]	None	Netherlands	Registry	2010–2020	88	ns	8072	8072	All	Yes	Yes	65.90%	Mean 66 (range 29–92)	Categorical	Hospital only	Yes	<13/year, 13–24, >24
Yapp et al. 2021 [5]	None	Scotland	Registry	1998–2019	71	ns	8894	8301	All	Yes	Yes	53.60%	Median 70 (IQR 63–76)	Categorical/continuous	Hospital and surgeon	Yes	<17/year, 17–27, 28–46, >46
Lindberg‐Larsen et al. 2016 [11]	Yes	Denmark	Registry	2011–2013 (follow‐up until 2015)	25	ns	320	305	Infection	No	No	43.90%	Categorised	Categorical	Hospital only	No	≤30 in 2 years, >30 in 2 years
Lindberg‐Larsen et al. 2014 [13]	Yes	Denmark	Registry	2009–2011	39	ns	1218	1165	Aseptic	No	No	60.40%	Mean 65.0 (range 27–94)	Categorical	Hospital only	Yes	<10/year, 10–30/year and >30/year
Blackburn et al. 2023 [15]	None	USA	Single healthcare network database	2016–2021	ns	8	568 patients	568	All	No	No	54.50%	66 years (range 45–86)	Continuous	Surgeon only	Yes	Not categorised
Klasan et al. 2021 [12]	None	New Zealand National Joint Registry	Registry	1999–2015	ns	ns	1720	1720	Aseptic	No	No	48.80%	Mean 66.3 (SD 9.6)	Categorical	Surgeon	Yes	<5 overall, ≤5/year, ≥5/year
Roof et al. 2021 [8]	No funding	USA	Institutional Database	2016–2019	2	24	308	287	Aseptic	No	Yes	Categorised	Categorised	Centiles	Surgeon only	Yes	>40% percentile HV; ≥19/year HV, <19 LV

Abbreviations: IQR, interquartile range; SD, standard deviation.

### Exposure calculation

Eight out of ten studies used annual surgeon or hospital volume in their definitions of caseload. Most studies aggregated annual volumes based on average yearly procedural numbers for each hospital or surgeon. No studies presented a dynamic assessment of volume such as the procedural numbers in the 365 days prior to each index case for individual surgeons or hospitals. There was also substantial variation in the volume categories, which were chosen to define their subsequent scale of volume, and relatively few studies reported volume as a continuous variable (2 out of 10). Only one of these studies referred to non‐linear relationships of continuous variables and used flexible modelling methods to account for this (e.g., restricted cubic splines).

### Primary and secondary outcomes

A summary of the study results and evidence certainty are summarised in Table 2. For a detailed breakdown of individual study results for each outcome assessed, refer to Supporting Information S2. The association of hospital volume with re‐revision was reported in five studies [14, 20, 21, 38, 39]. Due to the differences in outcome measures, these studies were analysed separately and as such, we were limited in the extent to which the results could be pooled, and a SWiM approach was used. For studies assessing re‐revision risk in the early post‐operative period, there was inconsistency in the results across studies. In the larger adjusted study representing 94.1% (23,644/25,144) of patients, higher hospital volume was associated with a reduction in re‐revision rate [14]. The results of the two studies reporting on re‐revision risk at any time point showed inconsistent results between studies. Both studies were equally weighted in populations, with one study having a higher event rate than the other. Similarly adjusted estimates for well‐known confounding factors such as age, gender, co‐morbidity, indication for revision, component revised and previous revision knee arthroplasty were reported in both studies.

**Table 2:** Summary of findings and certainty of evidence (GRADE).

Certainty assessment							Study event rates	Effect	Certainty	Importance
No. of studies	Study design	Risk of bias	Inconsistency	Indirectness	Imprecision	Other considerations	(*n*/*N*) (%)			
**Hospital re‐revision within 2 years (assessed with: odds ratio)**										
3	Non‐randomised studies	Serious^a^	Not serious^b^	Serious^c^	Not serious^d^	None^e,f^	2100/23,964 (8.8%)	Results were inconsistent across studies; However higher hospital volume was associated with lower re‐revision rates in the larger study (94% patients) with lower risk of bias. Dose–response relationship not tested in absence of raw data for one study	⊕◯◯◯Very low^a,b,c,d,e,f^	CRITICAL^ba^
**Hospital re‐revision at any point in time**										
2	Non‐randomised studies	Serious^g^	Serious^h^	Not serious^i^	Serious^j^	None	2100/16,966 (12.4%) Excludes study without raw data	Results were inconsistent across studies; both analyses were adjusted with equal numbers of patients and moderate risk of bias.	⊕◯◯◯Very low^g,h,i,j^	CRITICAL^az^
**Hospital mortality within 1 year**										
4 (3 in DRMA)	Non‐randomised studies	Serious^k^	Serious^l,m^	Very serious^n^	Very serious^o,p,q^	No strong evidence of a dose–response gradient^r,s^	395/36,389 (1.1%)	Results are inconsistent across studies. The largest study representing 65% of the population reported statistically insignificant results in the highest volume hospitals.	⊕◯◯◯Very low^k,l,m,n,o,p,q,r,s^	CRITICAL^bb^
**Hospital adverse medical events up to 1 year**										
3	Non‐randomised studies	Serious^t^	Not serious^u,v^	Serious^w^	Serious^x,y^	Dose–response gradient^a,az^	1022/35,524 (2.9%)	There is evidence of a dose–response association for hospitals with annual volumes above 56 revisions. Below this volume, the results are inconsistent. Two out of three studies report adjusted results and measure outcomes in similar time periods.	⊕⊕◯◯Low^aa,t,u,v,w,x,y,z^	CRITICAL^bc^
**Hospital length of stay (Admission to discharge)**										
1	Non‐randomised studies	Very serious^ab^	Not serious^ac^	Serious^ad^	Serious^ae^	Publication bias strongly suspected^af^	*N* = 1165	Lower‐volume hospitals were associated with shorter lengths of stay following a partially adjusted analysis	⊕◯◯◯Very low^ab,ac,ad,ae,af^	IMPORTANT^bd^
**Hospital readmission within 90 days (follow‐up: 90 days)**										
1	Non‐randomised studies	Very serious^ag^	Not serious^ac^	Serious^ah^	Serious^ai^	Publication bias strongly suspected^af^	120/1218 (9.9%)	Higher hospital volume was not associated with lower rates of readmissions within 90 days	⊕◯◯◯Very low^ac,af,ag,ah,ai^	IMPORTANT
**Surgeon re‐revision at any point in time**										
2	Non‐randomised studies	Serious^aj^	Not serious^ak^	Not serious^al^	Not serious^am^	None^an,ao^	208/1720 (12.1%)	Higher surgeon volume was not associated with lower cumulative risk of re‐revision	⊕◯◯◯Very low^aj,ak,al,am,an,ao^	CRITICAL
**Surgeon length of stay (Admission to discharge) (follow‐up: range 1–2 years)**										
1	Non‐randomised studies	Very serious^ag^	Not serious^ac^	Very serious^ap^	Very serious^aq^	All plausible residual confounding would suggest spurious effect, while no effect was observed^ar^	169 patients undergoing full revisions only	No significant differences found between higher surgeon volume and length of stay	⊕◯◯◯Very low^ac,ag,ap,aq,ar^	IMPORTANT
**Surgeon readmission within 90 days (follow‐up: 90 days)**										
1	Non‐randomised studies	Very serious^ag^	Not serious^ac^	Very serious^ap^	Very serious^as^	All plausible residual confounding would suggest spurious effect, while no effect was observed^at^	15/169 (8.9%) inclusive of those undergoing only full revision procedures	No significant differences found between higher surgeon volume and lower rates of all‐cause readmission at 90 days	⊕◯◯◯Very low^ac,ag,ap,as,at^	IMPORTANT
**Surgeon operating times (incision and wound closure)**										
1	Non‐randomised studies	Very serious^ag^	Not serious^ac^	Very serious^ap^	Very serious^aq^	All plausible residual confounding would suggest spurious effect, while no effect was observed	*N* = 308 includes those undergoing partial and full revision procedures	Mean operating time was found to be significantly shorter for high‐volume surgeons	⊕◯◯◯Very low^ac,ag,ap,aq^	IMPORTANT
**Surgeon re‐revision within 1 year (follow‐up: range 1–5 years)**										
1	Non‐randomised studies	Very serious^ag^	Not serious^ac^	Very serious^ap^	Very serious^au^	All plausible residual confounding would suggest spurious effect, while no effect was observed	21/169 (12.4%) inclusive of only those undergoing full revision procedures	High‐volume surgeons had significantly lower rates of reoperations or revisions within 1 year	⊕◯◯◯Very low^ac,ag,ap,au^	CRITICAL
**Surgeon Oxford Knee Score at 6 months**										
1	Non‐randomised studies	Very serious^ag^	Not serious^ac^	Serious^av^	Very serious^aw^	All plausible residual confounding would suggest spurious effect, while no effect was observed^ar^	*N* = 1720	Higher surgeon volume was not associated with improved Oxford Knee Scores at 6 months post‐operatively	⊕◯◯◯Very low^ac,ag,ar,av,aw^	CRITICAL
**Surgeon PROMIS‐PF‐10a**										
1	Non‐randomised studies	Very serious^ag^	Not serious^ac^	Very serious^ax^	Very serious^ay^	All plausible residual confounding would suggest spurious effect, while no effect was observed	*N* = 569	Higher surgeon volume was not associated with poorer MCID achievement rates	⊕◯◯◯ Very low^ac,ag,ax,ay^	CRITICAL

*Note*: Explanations for the superscript letters are provided below.

a. Overall risk of bias was serious in two studies and moderate in one study; however, the moderate study represented 94% of the total number of patients (23,644/25,114), decision to downgrade to serious.

b. The largest study, contributing 94% of the patients, shows a consistent effect. The smaller studies, which contribute only 6% of patients, show no effect. These differences are likely due to smaller sample sizes with wider confidence intervals seen in the smaller studies representing insufficient power in the smaller studies. Therefore, we did not downgrade for inconsistency.

c. While the target population involves revision knee replacement patients. The largest study involves patients from an administrative database, representative of only 30% of the German population. The population only includes aseptic revisions

d. Most of the evidence comes from large studies with narrow confidence intervals and sufficient sample sizes. Although smaller studies showed wider confidence intervals, they contributed less than 25% of the total participants. Therefore, we did not downgrade for imprecision.

e. While there is serious reporting bias in the two smaller studies, the larger study which represents the majority of patients and events reports adjusted estimates transparently.

f. Only one study adjusts its results for important confounding variables such as age, gender, co‐morbidities, and component revised and previous revision knee arthroplasty. However, this study represents the overwhelming majority of patients. Therefore, the decision not to downgrade.

g. Two studies equally weighted, overall risk of bias was moderate for both studies. Decision to downgrade to serious.

h. Different trends are seen across both studies. One study reported non‐significant effects with the other reporting significant effects in support of a hospital volume–outcome relationship. Therefore decision to downgrade to serious.

i. Both studies recruit patients with revision knee replacements from national registry data. With similar age, gender and co‐morbidities. The same outcome is assessed over a slightly different median follow‐up. Decision not to downgrade.

j. One study reported a significant effect in favour of higher volume hospitals with relatively narrow confidence intervals. One study showed no evidence of a significant effect with narrow confidence intervals. Despite individual precision, there is overall imprecision in the results. Both are appropriately adjusted for confounding factors.

k. Overall risk of bias was reported to be serious in three studies and moderate in one study. Despite this, the study graded as moderate contributes 23,644/36,389 (65%), representing the majority, which reduces the impact of the serious bias in the other studies. Decision to downgrade to serious.

l. Following the pooling of results for the dose–response meta‐analysis, the *I* ^2^ statistic was 62.9% with a statistically significant *p* value suggesting between‐study heterogeneity. There was no statistically significant dose–response relationship following modelling of dose as a non‐linear variable.

m. The largest study representing 65% of the population has inconsistent results across volume categories. With significantly lower mortality reported in the middle two volume categories and non significant mortality reported in the highest hospital volume categories. The remaining studies despite showing a trend do not show evidence of significant effect. There is inconsistency between the results.

n. Two studies present data on infected revision knee arthroplasty patients, while the largest study (65% of the population) reports on only a non‐infected patient cohort. One further study reports on all revision knee arthroplasty patients without adjusting for indication which is a key confounder. Pooling these heterogeneous populations into a dose‐dependent meta‐analysis introduces significant indirectness, as the results are not directly applicable to any specific subgroup. Even if the larger study contributes the most weighting, it only applies to a non‐infected population. Given the clinical and methodological differences across the studies, the evidence was downgraded by two levels for very serious indirectness.

o. Mortality was a rare event, there were 36,389 patients at risk in the pooled studies with only 395 deaths.

p. When results are pooled assuming a nonlinear relationship and modelled with restricted cubic splines, there is imprecision in the results below a hospital volume threshold of 56, where confidence intervals overlap 1.0.

q. The largest study reports narrow confidence intervals for some hospital volume ranges and wide confidence intervals for the highest volume category suggesting imprecision. The remaining three studies, despite showing a trend, report non‐statistical differences with wide confidence intervals. Two studies present unadjusted analyses. There is individual and combined imprecision in the results.

r. Reporting bias was noted to be low in the largest study representative of more than 65% of the population.

s. The largest study contributing a greater proportion of the population (65% of population) represents an adjusted analysis for age, gender, comorbidity, previous revision knee arthroplasty, prosthetic component revised, indication for revision but not surgeon volume. One study presents a partially adjusted analysis. The other two studies report an unadjusted analysis. Decision not to downgrade as larger study contributes the most effect, however unadjusted results are noted in the other studies.

t. Overall risk of bias was moderate in two studies and serious in one study. Since studies with moderate risk of bias accounted for >75% of patients (26,630/35,524), we downgraded the risk of bias to serious only.

u. Following the pooling of results, the *I* ^2^ statistic is 33.6%, and the univariate Cochran *Q* test for residual heterogeneity had a non‐significant *p* value of 0.21. There was a statistically significant relationship between higher hospital volume and lower odds of post‐operative complications.

v. Same trend (consistent decrease with increasing hospital volumes) for studies accounting for 32538/35524 of patients; smaller studies with inconsistent effects.

w. In the largest study (23,644/35,524), the population was taken from a subset of revision knee arthroplasty patients to include only non‐infected revisions from a population representative of 30% of their national population. As the weighted majority study, there was a decision to downgrade. The remaining two studies equally take separate subsets of a revision population (an infected cohort and all revision knee arthroplasty patients), and a dose‐dependent meta‐analysis requires a homogenous population. The outcome of the largest study was a risk of adverse in‐hospital events. This outcome was shared with another study. However, the third study measured the risk of DVT/PE within 1 year, which introduced further bias in the pooling of results.

x. When results are pooled assuming a nonlinear relationship and modelled with restricted cubic splines, there is imprecision in the results below a threshold of 57 where confidence intervals overlap 1.0.

y. The largest study reported non‐significant results for the largest hospital volume group despite showing a statistical difference for the middle two volume groups. Suggesting imprecise results. As this was the majority, decision to downgrade to serious. However, the other two studies reported no statistical difference across volume groups.

z. Reporting bias was low in two studies representing the majority of patients

aa. Age, gender, and co‐morbidity were adjusted for in two studies. This represented most patients in the pooled results 26,630/35,524, decision not to downgrade, although the other study was noted to provide no adjusted estimates.

ab. Only one study for this outcome was found and overall risk of bias was found to be serious. Decision to downgrade to very serious.

ac. Only one study was identified for this outcome comparing two volume groups. As consistency evaluates variability across multiple studies, it could not be assessed in this case. Therefore, a decision was made not to downgrade.

ad. Single study only including patients undergoing revision knee arthroplasty were recruited from a large national registry; however, they focussed on a subset of non‐infected patients. The outcome was length of stay and is formally mandated for cost reimbursement purposes, which provides an accurate outcome measure. Decision to downgrade to serious.

ae. Single study suggests hospital length of stay is longer for higher volume hospitals, although wide confidence intervals are seen. Raising concern about study imprecision and as a result has been downgraded to serious.

af. There is serious reporting bias, as evidenced by the lack of reporting of patients at risk and those with events in each volume group tested.

ag. This was a single study and the overall risk of bias was found to be serious. Therefore, the decision to downgrade evidence to very serious.

ah. Single study only including patients undergoing revision knee arthroplasty were recruited from a large national registry; however, they focussed on a subset of non‐infected patients, 1165. Small number of patients only. The outcome was readmission within 90 days of stay and is formally recorded providing an accurate outcome measure. Decision to downgrade to serious as not representative.

ai. Single study reporting no statistical effect, but wide confidence intervals are given suggesting imprecision in the study results.

aj. Risk of bias moderate in largest study and serious in smaller study. Larger study represents 84% of the total number of patients, therefore decision to downgrade to serious only.

ak. Similar trend seen in both studies indicating non‐significant effects.

al. The larger study recruited patients from a national joint registry. This included all revision knee arthroplasty patients. The outcome was re‐revision and this was collected following a median follow‐up of 6.2 years. Decision not to downgrade.

am. The total number of patients included in the studies is 10,614. In the larger study representing 8894 patients the confidence intervals are very narrow. The smaller study does not report raw event data for respective volume categories but has much wider confidence intervals. As the larger study represents most of the patients, the decision was made not to downgrade.

an. The larger study reports an adjusted analysis for the key confounding variables representing most patients. The smaller study does not present an adjusted estimate beyond indication for revision and previous revision arthroplasty. Therefore, borderline to not serious publication bias.

ao. The larger study representing most patients adjusted for all known confounders.

ap. Single study recruiting revision knee arthroplasty patients from an institutional database, excluding infections and revisions from partial knee replacements. Small number of patients only 169 patients. Decision to downgrade to very serious.

aq. Single study, results are unadjusted. Mann–Whitney *U* test used reporting non‐significant *p* value. However, no study effect estimated and 95% confidence intervals were reported. The study is also likely underpowered to detect meaningful change. Downgraded to very serious for precision.

ar. Study results are unadjusted for any confounding variables.

as. Single study, unadjusted analysis presented, non‐significant *p* values although very very wide confidence intervals. Imprecision downgraded to very serious.

at. Estimates were not adjusted for any known confounding factors.

au. Single study results, across two surgeon volume categories. Non‐significant effects found, but very wide confidence intervals. The estimates were unadjusted for known confounders.

av. 1,720 patients with revision knee arthroplasty recruited from the New Zealand National Joint Registry is voluntary but has been shown to have good compliance and is likely representative of all revision knee activity in the population. Despite this, their population subset excluded infection, and the results are less generalisable to all revision knee arthroplasty patients. The outcome was Oxford Knee Score (A validated instrument in the assessment of knee function in a revision knee arthroplasty population). Decision to downgrade to serious.

aw. The estimates presented are unadjusted for any known confounding factors. Also, these estimates, while not reaching statistical significance, have very large confidence intervals, raising concerns about the precision of the study results. Downgraded to very serious.

ax. Only 568 patients undergoing revision knee arthroplasty patients were recruited from a single healthcare network consisting of two academic medical centres and three community hospitals. The PROMIS‐PF Scale is not available as a raw continuous scale. Instead, it has undergone a complex transformation and the outcome is defined by achievement rates of minimally important clinical differences for worsening scores. This has not been validated in a revision knee arthroplasty population. Decision to downgrade to very serious indirectness.

ay. Single study presents unadjusted estimates excluding potentially known confounding variables and reports wide confidence intervals. Downgraded to very serious precision.

Two studies investigated the association between surgeon volume and re‐revision at any point using longitudinal registry data [17, 39]. Both studies have a high volume of patients with 8894 and 1720, respectively. The larger study represents over 84% of the patients and presented an adjusted analysis, considering demographic characteristics such as age, gender and co‐morbidities in addition to clinical characteristics such as type of component revised and previous revision knee arthroplasty [39]. This study also adjusted for the effect of hospital volume on re‐revision rates. There was no association between surgeon volume and re‐revision at any point in time. Only one study investigated the relationship between surgeon volume and early re‐revision within 1 year. Roof et al. suggested higher volume surgeons achieve significantly lower rates of reoperations [27]. However, a small number of patients were recruited from a single institution, and the overall risk of bias was found to be serious.

**Figure 2:**
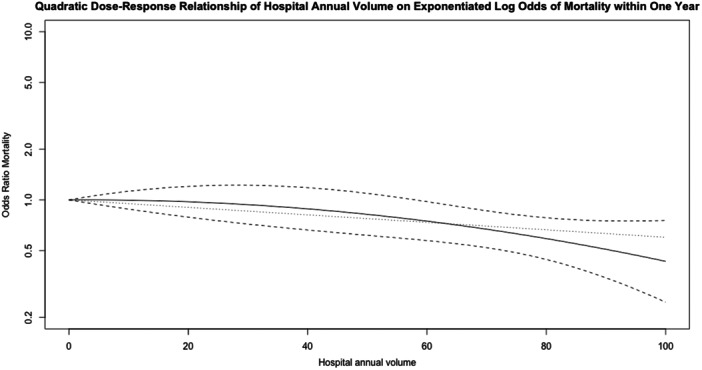
Dose–Response relationship for hospital volume and odds ratio (OR) of mortality up to 1 year after RevKR. The black solid line in the middle indicates the OR, and the upper and lower black dotted lines indicate a 95% CI. The blue dotted line at the bottom indicates the OR of the linear model. The risk in the quadratic model is slightly different from the risk in the linear model. The quadratic model has a non‐significant *p* value of 0.34 indicating there is no strong evidence of a dose–response relationship. The *I* ^2^ statistic is 62.8% indicating moderate residual heterogeneity. CI, confidence interval; RevKR, revision total knee replacement.

Four studies investigated the association between hospital volume and mortality up to 1 year representing 36,389 patients [14, 21, 33, 39]. The pooled crude mortality rate across studies was very rare, affecting only 1.1% (395/36,389). The outcome timeframe varied considerably between studies with two studies reporting mortality within 90 days, one study reporting mortality within 1 year and a further study reporting inpatient mortality only. There was no strong evidence of a dose–response relationship (Figure 2). The largest study used an adjusted analysis and contributed 23,644/36,389 (65%) of the total patients with only a moderate overall risk of bias. However, in this study, there were inconsistent results across hospital volume categories.

**Figure 3:**
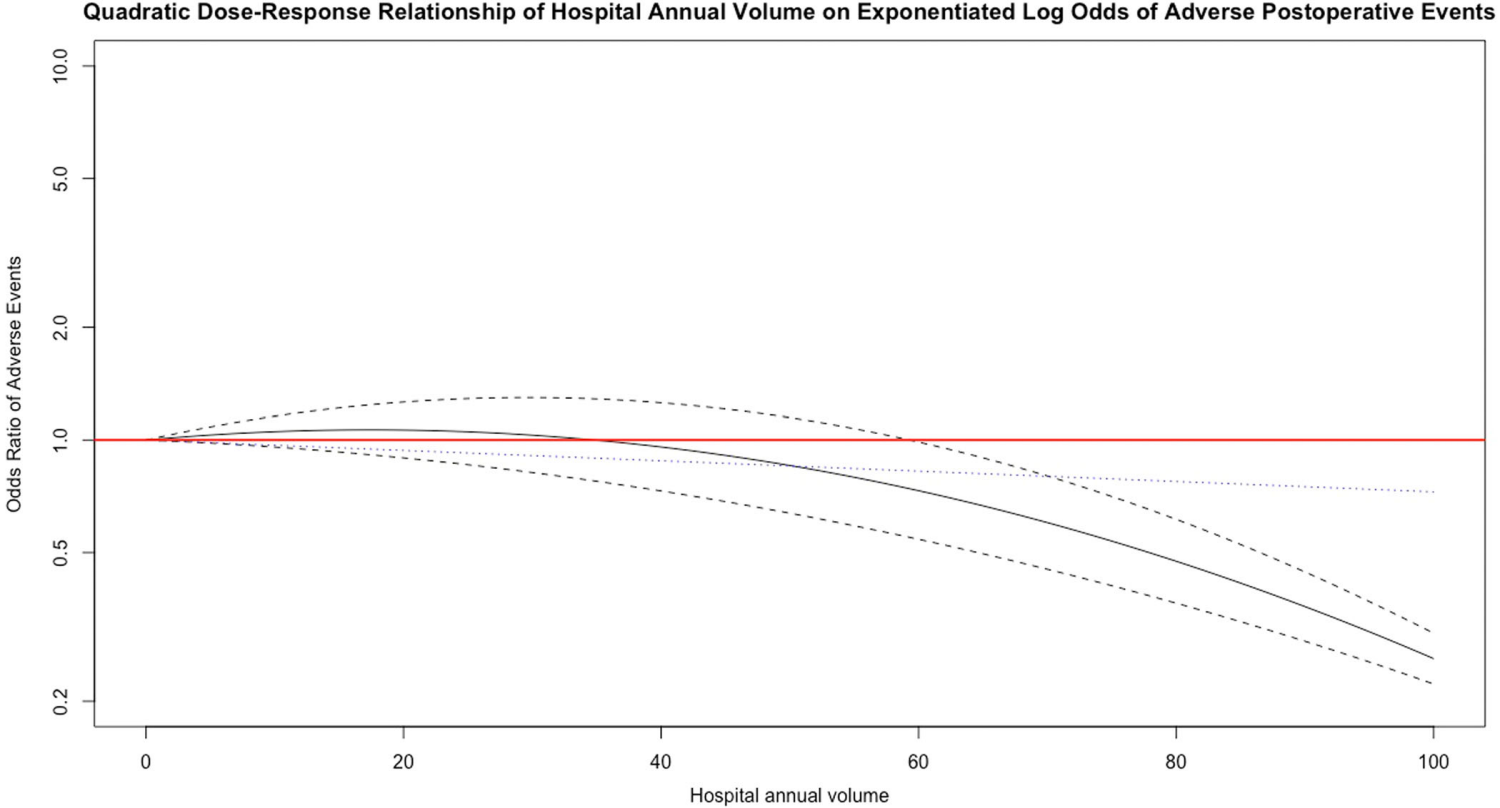
Dose–response relationship for hospital volume and rates of adverse post‐operative events up to 1 year after RevKR. The black solid line in the middle indicates the odds ratio (OR) of adverse post‐operative complications, and the upper and lower black dotted lines indicate a 95% CI. The blue dotted line at the bottom indicates the odds ratio from the linear model. The quadratic model has a significant *p* < 0.05, indicating when modelled as a quadratic term, hospital volume is related to odds of post‐operative complications. Higher hospital volumes are associated with lower odds of complications, For annual hospital volumes ≥60, the OR and associated 95% CI are below 1.0. The *I* ^2^ statistic is 33.6% indicating low residual heterogeneity. CI, confidence interval; RevKR, revision total knee replacement.

Three studies investigated the association between hospital volume and adverse post‐operative events [10, 14, 39]. The overall complication rate was 2.9% (1022/35,524). Adverse events had similar definitions in two studies, which included respiratory, renal, cardiac and cerebrovascular complications in addition to pulmonary embolism, which occurred during the patient's hospital stay. One study defined adverse events as the presence of deep venous thrombosis or pulmonary embolism only within 1 year. Figure 3 shows higher hospital volume was statistically associated with reduced odds of complications, particularly in hospitals performing 60 or more annual RevKRs. There was no evidence of between‐study residual heterogeneity in the pooling of these results, with an *I* ^2^ statistic of 33.6%. However, these data include both adjusted and unadjusted results. Higher hospital volume may be associated with lower odds of post operative complications, however the confidence in the cumulative evidence is low.

Only two studies tested the association between surgeon volume and outcomes related to improvement in knee function and pain reduction (*n* = 2289) [3, 17]. Both studies reported different outcomes and measurement intervals and were subsequently analysed separately. Oxford Knee Score has been shown to demonstrate good measurement properties in a cohort of patients undergoing RevKR [28]. However, patients in the PROMIS‐PF group were classified based on patients reaching Minimal Clinically Important Difference for Worsening (MCID‐W). This has yet to be validated in a RevKR population. Both studies suggest surgeon volume is unrelated to PROMs. However, neither estimate adjusts for any potential confounding factors such as age, gender, co‐morbidity or baseline functional scores. There may be no association between surgeon volume and patient‐reported outcomes; however, the certainty of this evidence is very low.

There was a scarcity of evidence for the following outcomes: hospital volume and readmissions within 90 days; surgeon volume and readmissions within 90 days; hospital volume and length of inpatient stay; surgeon volume and length of inpatient stay and surgeon volume and operating times. No studies investigated the association between hospital or surgeon volume and cost efficiency.

## DISCUSSION

This review identified 10 papers reporting the relationship between number of procedures performed and outcomes following RevKR [3, 10, 14, 17, 20, 21, 27, 33, 38, 39]. Considering the newer evidence to date, the overall literature is more unclear and inconsistent than previously reported. The confidence in the cumulative evidence was very low due to the serious risk of bias driven by the observational study designs, population heterogeneity, unadjusted regression estimates for known significant confounders and varying time points in outcome assessment. The scarcity of evidence pertaining to this research topic has again been highlighted with several outcomes limited to only one study.

Understanding the volume‐outcome relationship is important, particularly as recent evidence has been used to plan optimal service delivery models in the UK [16]. Over the last decade, there have been several studies published in the literature considering low volume, less specialist practice [4, 15]. There are also several known confounders that influence outcomes in RevKR and historically these have not been included in the modelling of outcomes [1, 9]. It is reassuring that more recent registry‐based volume/outcome studies have adjusted for these in their estimates.

The primary outcome was re‐revision and its association with hospital and/or surgeon volume was reported by five [14, 20, 21, 38, 39] and three [17, 27, 39] studies, respectively. However, differences in the reporting of re‐revision rates prevented the pooling of estimates. Re‐revision was either measured at a fixed time point typically the presence of re‐revision at 1 year or 2 years [14, 20, 21] or it was measured as part of a survival analysis as the rate of re‐revision at any point in time [38, 39]. Early re‐revision may be more relevant in the context of assessing the quality of the surgical provision. Re‐revision at longer follow‐up times may be biased by unrelated factors such as the haematogenous spread of infection. The evidence was inconsistent for studies which measured re‐revision rate and hospital volume in this way. Similarly, surgeon volume was found to be unrelated to the overall re‐revision rate, yet Roof et al. [27] showed statistically fewer re‐revisions amongst higher volume surgeons at 1 year. However, this study was seriously biased due to the selection of participants and in the estimation of effects. Future studies should use early re‐revision as their primary outcome so that their data can be used more effectively for further research.

The mortality rate was found to be very low, which was consistent with recent epidemiological studies in RevKR [30]. Mortality was only used as an outcome for hospital volume and was most often measured within 90 days. There was no evidence of a dose–response relationship with hospital volume and mortality. The rarity of the outcome could partly explain the inconsistencies observed within the included studies. However, as with all similarly designed studies, the absence of a power calculation in study designs is not acknowledged in their limitations, and we suggest this should be standard practice.

RevKR is often associated with increased morbidity and higher rates of complications than primary TKR [11]. A dose–response relationship was found between hospital volume and adverse post‐operative events, with annual hospital volumes of ≥60 associated with fewer complications. However, due to bias in the measurement of complications, the statistical confidence in this estimate is low.

Outcomes related to function and quality of life were poorly reported. Only two studies investigated the association between surgeon volume and PROMs [3, 17]. Both studies reported a knee‐specific PROM without the inclusion of a generic health‐related quality of life measure. This is not surprising and is consistent with previously reported studies investigating outcomes following RevKR, but it should be recognised that quality of life measures should accompany future research reporting functional knee scores [22]. Outcomes measures were Oxford Knee Scores and PROMIS‐PF 10a scores and both studies failed to show an effect. However, both studies did not adjust for confounding factors such as age, gender, co‐morbidities, indication and baseline PROMs for revision despite these factors being associated with lower post‐operative PROM scores [29].

### Limitations

The definition of volume is consistently described in the literature, with average annual volume typically informing volume calculations. However, most studies categorise volume according to either the spread of data or use pre‐defined categories based on previous literature. Categorisation of continuous predictors leads to loss of information, and it is typically not advised unless there is a clinically meaningful cut‐off that is used to partition the data. Only two studies [38, 39] analysed annual volume as a continuous variable, with one recognising the non‐linear relationship during the modelling of outcomes [39]. An attempt was made to pool studies categorising volume as part of a DRMA, but this was only possible for two outcomes, which had a minimum of three studies for analysis. Despite pooling results in a DRMA, both adjusted and unadjusted study estimates were included which could bias the pooled estimates. Study estimates were also pooled from cohorts of septic and non‐septic patients. Septic populations are generally more prone to complications such as re‐revision, mortality, and adverse medical complications [32]. Sensitivity analysis, including only adjusted estimates or studies evaluating patients with similar reasons for revision, was not possible due to the paucity of studies for each reported outcome. Another potential source of bias was the estimation of ORs and 95% CIs when not directly reported by a study, particularly when the risk of the event was rare, potentially leading to imprecise estimates.

The paucity of studies for the polled analysis was driven to a large extent by a lack of standardisation in the reported outcomes between studies. This was seen both in terms of the definition of the outcome and the time point where the measurement was taken. This is important, particularly as RCTs are not feasible, and we rely on observational longitudinal data to draw conclusions. Given the rarity of outcomes in RevKR, we acknowledge the difficulty in effectively powering a volume–outcome study, particularly as earlier registry data are often less valid as a reflection of current practice. This further argues the case that subsequent volume–outcome studies should report the same outcomes at the same time points to enable their data to be used more effectively in future research.

Understanding the volume‐outcome relationship is important particularly when recommendations of minimum activity thresholds for surgeons and hospitals have been introduced into routine clinical practice [5]. These are the minimum number of annual procedures a hospital or surgeon may perform. This threshold has been set at 30 annual revisions for hospitals and 15 annual revisions for surgeons. Such recommendations were based on the existence of evidence in support of a volume‐outcome relationship. However, variations in the definitions of high and low volume of the heterogeneity in outcome measures and bias in effect estimates make it difficult to draw inferences from the most recent literature. There may be an association between higher hospital volume and fewer adverse post‐operative events, but it remains clear, however, there is an urgent need for future research to investigate this relationship and provide evidence‐based estimates as to what the optimum surgeon and hospital volumes should be for RevKR procedures. This work will be pertinent to the recommendations of specialist orthopaedic societies [5] on minimum activity targets for surgeons and hospitals.

## CONCLUSION

There is an unclear and inconsistent relationship between surgeon and hospital volume and outcomes. Inconsistency in reporting outcomes, population heterogeneity and a lack of adjustment for confounding in estimates make it difficult to provide any recommendations for clinical practice. Future research is needed to guide optimal service delivery models.

## AUTHOR CONTRIBUTIONS

All authors had full access to all the data in the study and had final responsibility for the decision to submit for publication. **Alexander H. Matthews**: Conceptualisation; methodology; funding acquisition; software; project administration; investigation; data curation; formal analysis; validation; visualisation; writing—original draft; writing—review and editing. **Tom Stringfellow**: Data curation; validation; writing—review and editing. **Hayley Redman**: Data curation; validation; writing – review and editing. **William K. Gray**: Conceptualisation; methodology; investigation; validation; supervision; writing—review and editing. **Jonathan P. Evans**: Conceptualisation; supervision; writing—review and editing. **Jonathan T. Evans**: Supervision; writing—review and editing. **Sarah E. Lamb**: Supervision; writing—review and editing. **Timothy Briggs**: Funding acquisition; Supervision; Writing—review and editing. **Andrew Price**: Conceptualisation; funding acquisition; supervision; writing—review and editing. **Andrew Toms**: Conceptualisation; Funding acquisition; Supervision; Writing—review and editing.

## CONFLICT OF INTEREST STATEMENT

The authors declare no conflicts of interest.

## ETHICS STATEMENT

Ethical approval was not required for this study.

## Supporting information

Supporting information.

Supporting information.

Supporting information.

## Data Availability

The data sets generated and analysed in the current study were generated from publicly available data sources.
