# Supplementary material for: Low confidence in the cumulative evidence for the existence of a volume–outcome relationship after revision total knee replacement: A systematic review and meta‐analysis
Source: Knee Surg Sports Traumatol Arthrosc. 2025 Mar 11;33(7):2555–70. doi: 10.1002/ksa.12641 (PMC12205428; doi:10.1002/ksa.12641)
Supplement: Supplementary file 2 — Supporting information. [file KSA-33-2555-s001.docx]

Supplementary material S1

**Table 1: Electronic search terms and syntax used in search strategy for MEDLINE and EMBASE via Ovid Silver platter**

**Revision Knee replacement**

Revision ADJ3 knee replacemen* (includes replacement and replacements

Revision ADJ3 knee arthroplast* (Includes arthroplasty and arthroplaties)

Conversion ADJ3 knee replacemen*

Conversion ADJ3 knee arthroplast*

Debridement and Implant Retention adj3 Knee

DAIR adj3 Knee

Revision adj3 TKR

Revision adj3 UKR

Revision adj3 TKA

Revision adj3 UKA

RTKR

RTKA

Conversion adj3 TKA

Conversion adj3 TKR

Conversion adj3 UKR

Conversion adj3 UKA

Conversion adj3 RTKA

Secondary ADJ patella ADJ resurfacing

**AND**

**Volume**

("low volume surgeo*" OR "high volume surgeo*" OR "surgeon volum*" OR "high volume hospita*" OR "low volume hospita*" OR "volum*" OR “volum* ADJ3 outcom*” OR "hospital volum*" OR "hospital size" OR "center volum*" OR "center size" OR "centre size" OR "patient volum*" OR "provider volum*" " OR "surgical volum*" OR "procedure volum*" OR "procedural volum*" OR "facility volum*" OR "treatment volum*" OR regionali* OR centrali* OR caseload* OR workload* OR experience OR performance):ti,ab,kw

**NOT**

NOT ("Comment" [Publication Type] OR "Letter" [Publication Type] OR "Editorial" [Publication Type])
