# Supplementary material for: Low confidence in the cumulative evidence for the existence of a volume–outcome relationship after revision total knee replacement: A systematic review and meta‐analysis
Source: Knee Surg Sports Traumatol Arthrosc. 2025 Mar 11;33(7):2555–70. doi: 10.1002/ksa.12641 (PMC12205428; doi:10.1002/ksa.12641)
Supplement: Supplementary file 3 — Supporting information. [file KSA-33-2555-s003.docx]

# Supplementary material S2 – Study results, confounding factors and overall risk of bias by outcomes assessed

## Hospital volume & mortality

| **First author** | **Country** | **Time period** | **Publication year** | **Annual Volume categories** | **Volume midpoint** | **Follow up** | **Patients at risk** | **Numbers with event** | **Type** | **OR/HR** | **Ratio** | **L 95%CI** | **U 95%CI** | **Adjusted or unadjusted** | **Age, Gender, Comorbidity** | **Indication for revision** | **Component Revised** | **Previous Revision Knee Arthroplasty** | **Surgeon volume** | **Overall Risk of Bias** |
| --- | --- | --- | --- | --- | --- | --- | --- | --- | --- | --- | --- | --- | --- | --- | --- | --- | --- | --- | --- | --- |
| Halder et al | Germany | 2013 to 2017 | 2020 | <13 | 0 | within 90 days | 5763 | 58 | cumulative incidence | OR | 1.00 | 1.00 | 1.00 | Adjusted | yes | yes | yes | yes | no | Moderate |
| Halder et al | Germany | 2013 to 2017 | 2020 | 13 to 24 | 19 | within 90 days | 5445 | 44 | cumulative incidence | OR | 0.87 | 0.85 | 0.89 | Adjusted | yes | yes | yes | yes | no |  |
| Halder et al | Germany | 2013 to 2017 | 2020 | 25 to 52 | 39 | within 90 days | 6986 | 42 | cumulative incidence | OR | 0.60 | 0.58 | 0.62 | Adjusted | yes | yes | yes | yes | no |  |
| Halder et al | Germany | 2013 to 2017 | 2020 | >52 | 79 | within 90 days | 5450 | 27 | cumulative incidence | OR | 0.66 | 0.40 | 1.10 | Adjusted | yes | yes | yes | yes | no |  |
| Yapp et al | Scotland | 1998 to 2019 | 2021 | <17 | 0 | Within one year | 2344 | 34 | cumulative incidence | OR | 1.00 | 1.00 | 1.00 | Unadjusted | no | no | no | yes | no | Serious |
| Yapp et al | Scotland | 1998 to 2019 | 2021 | 17 to 27 | 22 | Within one year | 2229 | 21 | cumulative incidence | OR | 0.65 | 0.37 | 1.11 | Unadjusted | no | no | no | yes | no |  |
| Yapp et al | Scotland | 1998 to 2019 | 2021 | 28 to 46 | 37 | Within one year | 2107 | 26 | cumulative incidence | OR | 0.85 | 0.50 | 1.42 | Unadjusted | no | no | no | yes | no |  |
| Yapp et al | Scotland | 1998 to 2019 | 2021 | >46 | 64 | Within one year | 2214 | 25 | cumulative incidence | OR | 0.78 | 0.46 | 1.30 | Unadjusted | no | no | no | yes | no |  |
| Lindberg-Larsen et al | Denmark | 2011 to 2013 | 2016 | 0-15 | 0 | within 90 days | 165 | 12 | cumulative incidence | OR | 1.00 | 1.00 | 1.00 | Unadjusted | no | yes | no | no | no | Serious |
| Lindberg-Larsen et al | Denmark | 2011 to 2013 | 2016 | >15 | 30 | within 90 days | 155 | 1 | cumulative incidence | OR | 0.08 | 0.01 | 0.43 | Unadjusted | no | yes | no | no | no |  |
| Samuel et al | USA | 2007 to 2012 | 2022 | <17 | 0 | In hospital mortality | 1069 | 31 | cumulative incidence | OR | 1.00 | 1.00 | 1.00 | Adjusted | yes | yes | no | no | no | Serious |
| Samuel et al | USA | 2007 to 2012 | 2022 | 17 to 56 | 37 | In hospital mortality | 1610 | 58 | cumulative incidence | OR | 1.25 | 0.80 | 1.96 | Adjusted | yes | yes | no | no | no |  |
| Samuel et al | USA | 2007 to 2012 | 2022 | >56 | 76 | In hospital mortality | 852 | 16 | cumulative incidence | OR | 0.66 | 0.36 | 1.21 | Adjusted | yes | yes | no | no | no |  |

## Hospital volume and adverse postoperative events

| **First author** | **Country** | **Time period** | **Publication year** | **Annual Volume categories** | **Volume midpoint** | **Follow up** | **Patients at risk** | **Numbers with event** | **Type** | **OR/HR** | **Ratio** | **L 95%CI** | **U 95%CI** | **Adjusted or unadjusted** | **Age, Gender, Comorbidity** | **Indication for revision** | **Component Revised** | **Previous Revision Knee Arthroplasty** | **Surgeon volume** | **Overall Risk of Bias** |
| --- | --- | --- | --- | --- | --- | --- | --- | --- | --- | --- | --- | --- | --- | --- | --- | --- | --- | --- | --- | --- |
| Halder | Germany | 2013 to 2017 | 2020 | <13 | 0 | Adverse events during hospital stay | 5763 | 196 | ci | OR | 1.00 | 1.00 | 1.00 | Adjusted | Yes | Yes | Yes | Yes | no | Moderate |
| Halder | Germany | 2013 to 2017 | 2020 | 13 to 24 | 19 | Adverse events during hospital stay | 5445 | 180 | ci | OR | 1.00 | 0.99 | 1.00 | Adjusted | Yes | Yes | Yes | Yes | no |  |
| Halder | Germany | 2013 to 2017 | 2020 | 25 to 52 | 39 | Adverse events during hospital stay | 6986 | 189 | ci | OR | 0.87 | 0.87 | 0.88 | Adjusted | Yes | Yes | Yes | Yes | no |  |
| Halder | Germany | 2013 to 2017 | 2020 | >52 | 79 | Adverse events during hospital stay | 5450 | 131 | ci | OR | 0.85 | 0.63 | 1.15 | Adjusted | Yes | Yes | Yes | Yes | no |  |
| Feinglass | USA | 1993 to 1999 | 2004 | <7 | 0 | Adverse events during hospital stay | 1003 | 69 | ci | OR | 1.00 | 1.00 | 1.00 | Adjusted | Yes | no | no | no | no | Moderate |
| Feinglass | USA | 1993 to 1999 | 2004 | 7 to 14 | 11 | Adverse events during hospital stay | 1099 | 107 | ci | OR | 1.44 | 1.05 | 1.99 | Adjusted | Yes | no | no | no | no |  |
| Feinglass | USA | 1993 to 1999 | 2004 | >14 | 21 | Adverse events during hospital stay | 884 | 72 | ci | OR | 1.33 | 0.87 | 2.03 | Adjusted | Yes | no | no | no | no |  |
| Yapp | Scotland | 1998 to 2019 | 2021 | <17 | 0 | DVT/PE within one year | 2344 | 23 | ci | OR | 1.00 | 1.00 | 1.00 | Unadjusted | no | no | no | Yes | no | Serious |
| Yapp | Scotland | 1998 to 2019 | 2021 | 17 to 27 | 22 | DVT/PE within one year | 2229 | 18 | ci | OR | 0.82 | 0.44 | 1.52 | Unadjusted | no | no | no | Yes | no |  |
| Yapp | Scotland | 1998 to 2019 | 2021 | 28 to 46 | 37 | DVT/PE within one year | 2107 | 20 | ci | OR | 0.97 | 0.53 | 1.77 | Unadjusted | no | no | no | Yes | no |  |
| Yapp | Scotland | 1998 to 2019 | 2021 | >46 | 64 | DVT/PE within one year | 2214 | 17 | ci | OR | 0.78 | 0.41 | 1.46 | Unadjusted | no | no | no | Yes | no |  |

## Hospital volume and re-revision at any point in time

| **First author** | **Country** | **Time period** | **Publication year** | **Annual Volume categories** | **Volume midpoint** | **Follow up** | **Patients at risk** | **Numbers with event** | **Type** | **OR/HR** | **Ratio** | **L 95%CI** | **U 95%CI** | **Adjusted or unadjusted** | **Age, Gender, Comorbidity** | **Indication for revision** | **Component Revised** | **Previous Revision Knee Arthroplasty** | **Surgeon volume** | **Overall Risk of Bias** |
| --- | --- | --- | --- | --- | --- | --- | --- | --- | --- | --- | --- | --- | --- | --- | --- | --- | --- | --- | --- | --- |
| Van Rensch et al | Netherlands |  | 2023 | <13 | 0 | Median 3.7 yrs (0-13.7 range) | 2500 | 665 | Survival analysis | HR | 1.00 | 1.00 | 1.00 | Adjusted | yes | yes | yes | yes | no | Moderate |
| Van Rensch et al | Netherlands |  | 2023 | 13 to 24 | 19 | Median 3.7 yrs (0-13.7 range) | 2815 | 760 | Survival analysis | HR | 0.97 | 0.86 | 1.11 | Adjusted | yes | yes | yes | yes | no |  |
| Van Rensch et al | Netherlands |  | 2023 | >24 | 35 | Median 3.7 yrs (0-13.7 range) | 2757 | 758 | Survival analysis | HR | 0.94 | 0.83 | 1.07 | Adjusted | yes | yes | yes | yes | no |  |
| Yapp et al | Scotland |  | 2021 | <17 | 0 | Median FU 6.2 yrs (IQR 3.0 to 10.2) | 2,344 | 342 | Survival analysis | HR | 1.00 | 1.00 | 1.00 | Adjusted | yes | yes | yes | yes | yes | Moderate |
| Yapp et al | Scotland |  | 2021 | 17 to 27 | 22 | Median FU 6.2 yrs (IQR 3.0 to 10.2) | 2,229 | 244 | Survival analysis | HR | 0.81 | 0.68 | 0.96 | Adjusted | yes | yes | yes | yes | yes |  |
| Yapp et al | Scotland |  | 2021 | 28 to 46 | 37 | Median FU 6.2 yrs (IQR 3.0 to 10.2) | 2,107 | 206 | Survival analysis | HR | 0.71 | 0.59 | 0.85 | Adjusted | yes | yes | yes | yes | yes |  |
| Yapp et al | Scotland |  | 2021 | >46 | 64 | Median FU 6.2 yrs (IQR 3.0 to 10.2) | 2,214 | 165 | Survival analysis | HR | 0.60 | 0.49 | 0.73 | Adjusted | yes | yes | yes | yes | yes |  |

## Hospital volume and re-revision up to 2 years

| **First author** | **Country** | **Time period** | **Publication year** | **Annual Volume categories** | **Volume midpoint** | **Follow up** | **Patients at risk** | **Numbers with event** | **Type** | **OR/HR** | **Ratio** | **L 95%CI** | **U 95%CI** | **Adjusted or unadjusted** | **Age, Gender, Comorbidity** | **Indication for revision** | **Component Revised** | **Previous Revision Knee Arthroplasty** | **Surgeon volume** | **Overall Risk of Bias** |
| --- | --- | --- | --- | --- | --- | --- | --- | --- | --- | --- | --- | --- | --- | --- | --- | --- | --- | --- | --- | --- |
| Halder et al | Germany |  | 2020 | <13 | 0 | 1 yr | 5763 | 542 | cumulative incidence | OR | 1.00 | 1.00 | 1.00 | Adjusted | Yes | yes | yes | yes | no | Moderate |
| Halder et al | Germany |  | 2020 | 13 to 24 | 19 | 1 yr | 5445 | 523 | cumulative incidence | OR | 0.99 | 0.99 | 1.00 | Adjusted | Yes | yes | yes | yes | no |  |
| Halder et al | Germany |  | 2020 | 25 to 52 | 39 | 1yr | 6986 | 538 | cumulative incidence | OR | 0.78 | 0.78 | 0.78 | Adjusted | Yes | yes | yes | yes | no |  |
| Halder et al | Germany |  | 2020 | >52 | 79 | 1yr | 5450 | 403 | cumulative incidence | OR | 0.69 | 0.58 | 0.83 | Adjusted | Yes | yes | yes | yes | no |  |
| Lindberg-Larsen et al | Denmark |  | 2016 | 0-15 | 0 | Median follow up 2 years | 165 | 51 | cumulative incidence | OR | 1.00 | 1.00 | 1.00 | Unadjusted | no | yes | no | no | no | Serious |
| Lindberg-Larsen et al | Denmark |  | 2016 | >15 | 30 | Median follow up 2 years | 155 | 43 | cumulative incidence | OR | 0.86 | 0.53 | 1.39 | Unadjusted | no | yes | no | no | no |  |
| Lindberg-Larsen et al | Denmark | 2009 to 2011 | 2014 | <10 | 0 | Over 2 year study period, no minimum or median follow up | n/a | n/a | cumulative incidence | OR | 1.00 | 1.00 | 1.00 | Unadjusted | no | yes | no | no | no | Serious |
| Lindberg-Larsen et al | Denmark | 2009 to 2011 | 2014 | >=10 | 15 | Over 2 year study period, no minimum or median follow up | n/a | n/a | cumulative incidence | OR | 0.99 | 0.41 | 2.38 | Unadjusted | no | yes | no | no | no |  |

## Hospital volume and length of stay

| **First author** | **Country** | **Time period** | **Publication year** | **Annual Volume categories** | **Volume midpoint** | **Follow up** | **Patients at risk** | **Numbers with event** | **Type** | **OR/HR/Beta** | **Ratio** | **L 95%CI** | **U 95%CI** | **Adjusted or unadjusted** | **Age, Gender, Comorbidity** | **Indication for revision** | **Component Revised** | **Previous Revision Knee Arthroplasty** | **Surgeon volume** | **Overall Risk of Bias** |
| --- | --- | --- | --- | --- | --- | --- | --- | --- | --- | --- | --- | --- | --- | --- | --- | --- | --- | --- | --- | --- |
| Lindberg-Larsen | Denmark | 2009 to 2011 | 2014 | <10 | 0 | Admission and Discharge | n/a | n/a | cumulative incidence | Beta | 1 | 1 | 1 | Adjusted | Yes but not comorbidity | Yes | Yes | Yes | No | Serious |
| Lindberg-Larsen | Denmark | 2009 to 2011 | 2014 | >=10 | 15 | Admission and Discharge | n/a | n/a | cumulative incidence | Beta | 1.21 | 0.25 | 2.16 | Adjusted | Yes but not comorbidity | Yes | Yes | Yes | No |  |

## Hospital volume and emergency readmissions

| **First author** | **Country** | **Time period** | **Publication year** | **Annual Volume categories** | **Volume midpoint** | **Follow up** | **Patients at risk** | **Numbers with event** | **Type** | **OR/HR** | **Ratio** | **L 95%CI** | **U 95%CI** | **Adjusted or unadjusted** | **Age, Gender, Comorbidity** | **Indication for revision** | **Component Revised** | **Previous Revision Knee Arthroplasty** | **Surgeon volume** | **Overall risk of Bias** |
| --- | --- | --- | --- | --- | --- | --- | --- | --- | --- | --- | --- | --- | --- | --- | --- | --- | --- | --- | --- | --- |
| Lindberg-Larsen | Denmark | 2009 to 2011 | 2014 | <10 | 0 | Unplanned reoperations within 90 days | n/a | n/a | cumulative incidence | OR | 1 | 1 | 1 | unadjusted | no | yes | no | no | no | Serious |
| Lindberg-Larsen | Denmark | 2010 to 2011 | 2014 | >=10 | 15 | Unplanned reoperations within 90 days | n/a | n/a | cumulative incidence | OR | 1.16 | 0.7 | 1.92 |  |  |  |  |  |  |  |

## Surgeon volume and length of stay

| **First author** | **Country** | **Time period** | **Publication year** | **Annual Volume categories** | **Volume midpoint** | **Follow up** | **Patients at risk** | **Numbers with event** | **Type** | **OR/HR/Beta** | **Ratio** | **L 95%CI** | **U 95%CI** | **Adjusted or unadjusted** | **Age, Gender, Comorbidity** | **Indication for revision** | **Component Revised** | **Previous Revision Knee Arthroplasty** | **Hospital volume** | **Overall Risk of Bias** |
| --- | --- | --- | --- | --- | --- | --- | --- | --- | --- | --- | --- | --- | --- | --- | --- | --- | --- | --- | --- | --- |
| Roof | USA | 2016 to 2019 | 2021 | <19 | 0 | Admission and Discharge (minimum 1 year follow up) | 84 | 3.57 (2.69) Mean(SD) | cumulative incidence | Mann-Whitney | p = 0.066 | na | na | unadjusted | no | yes | yes | no | no | Serious |
| Roof | USA | 2016 to 2019 | 2021 | >=19 | 38 | Admission and Discharge (minimum 1 year follow up) | 85 | 2.98 (1.70) | cumulative incidence |  |  |  |  |  |  |  |  |  |  |  |

## Surgeon volume and Patient Reported Outcome Measures

| **First author** | **Country** | **Time period** | **Publication year** | **Annual Volume categories** | **Volume midpoint** | **Follow up** | **Patients at risk** | **Numbers with event** | **Type** | **OR/HR** | **Ratio** | **L 95%CI** | **U 95%CI** | **Adjusted or unadjusted** | **Age, Gender, Comorbidity** | **Indication for revision** | **Component Revised** | **Previous Revision Knee Arthroplasty** | **Hospital Volume** | **Overall Risk of Bias** |
| --- | --- | --- | --- | --- | --- | --- | --- | --- | --- | --- | --- | --- | --- | --- | --- | --- | --- | --- | --- | --- |
| Klasan | New Zealand | 1999 to 2015 | 2021 | <=5 | 0 | Oxford Knee Score at 6 months post operatively | n/a | n/a | cumulative incidence | Beta | 1 | 1 | 1 | unadjusted | no | yes | no | yes | no | Serious |
| Klasan | New Zealand | 1999 to 2015 | 2012 | >=5 | 10 | Oxford Knee Score at 6 months post operatively | n/a | n/a | cumulative incidence | Beta | 0.597 | -0.604 | 1.798 |  |  |  |  |  |  |  |
| Blackburn | USA | 2016 to 2021 | 2023 | n/a | n/a | PROMIS PF-10a Within 2.5years post operatively, MCID worsening score achievement rate | 569 | 123 | cumulative incidence | Beta | -0.02 | -0.042 | 0.0019 | unadjusted | no | no | no | no | no | Serious |

## Surgeon volume and re-revision at any time point

| **First author** | **Country** | **Time period** | **Publication year** | **Annual Volume categories** | **Volume midpoint** | **Follow up** | **Patients at risk** | **Numbers with event** | **Type** | **OR/HR** | **Ratio** | **L 95%CI** | **U 95%CI** | **Adjusted or unadjusted** | **Age, Gender, Comorbidity** | **Indication for revision** | **Component Revised** | **Previous Revision Knee Arthroplasty** | **Hospital volume** | **Overall Risk of Bias** |
| --- | --- | --- | --- | --- | --- | --- | --- | --- | --- | --- | --- | --- | --- | --- | --- | --- | --- | --- | --- | --- |
| Klasan | New Zealand | 1999 to 2015 | 2021 | <=5 | 0 | Re-revision at any point, no mean follow up given, cumulative revision risk | n/a | n/a | Survival analysis | HR | 1 | 1 | 1 | unadjusted | no | yes | no | yes | no | Serious |
| Klasan | New Zealand | 1999 to 2015 | 2021 | >=5 | 10 | Re-revision at any point, no mean follow up given, cumulative revision risk | n/a | n/a | Survival analysis | HR | 1.071 | 0.822 | 1.395 |  |  |  |  |  |  |  |
| Yapp | Scotland | 1998 to 2019 | 2021 | n/a | n/a | Re-revision at any point, based on 10 year survival | 8894 | 957 | Survival analysis | HR | 1.01 | 1 | 1.02 | Adjusted | yes | yes | yes | yes | yes | Moderate |

## Surgeon volume and re-revision up to one year

| **First author** | **Country** | **Time period** | **Publication year** | **Annual Volume categories** | **Volume midpoint** | **Follow up** | **Patients at risk** | **Numbers with event** | **Type** | **OR/HR** | **Ratio** | **L 95%CI** | **U 95%CI** | **Adjusted or unadjusted** | **Age, Gender, Comorbidity** | **Indication for revision** | **Component Revised** | **Previous Revision Knee Arthroplasty** | **Hospital volume** | **Overall Risk of Bias** |
| --- | --- | --- | --- | --- | --- | --- | --- | --- | --- | --- | --- | --- | --- | --- | --- | --- | --- | --- | --- | --- |
| Roof | USA | 2016 to 2019 | 2021 | <19 | 0 | re-revision within one year | 84 | 17 | cumulative incidence | OR | 1 | 1 | 1 | unadjusted | no | yes | yes | no | no | Serious |
| Roof | USA | 2016 to 2019 | 2021 | >=19 | 38 | re-revision within one year | 85 | 4 | cumulative incidence | OR | 0.2 | 0.05 | 0.56 | unadjusted |  |  |  |  |  |  |

## Surgeon volume and operating times

| **First author** | **Country** | **Time period** | **Publication year** | **Annual Volume categories** | **Volume midpoint** | **Follow up** | **Patients at risk** | **Numbers with event** | **Type** | **OR/HR** | **Ratio** | **L 95%CI** | **U 95%CI** | **Adjusted or unadjusted** | **Age, Gender, Comorbidity** | **Indication for revision** | **Component Revised** | **Previous Revision Knee Arthroplasty** | **Hospital volume** | **Overall Risk of Bias** |
| --- | --- | --- | --- | --- | --- | --- | --- | --- | --- | --- | --- | --- | --- | --- | --- | --- | --- | --- | --- | --- |
| Roof | USA | 2016 to 2019 | 2021 | <19 | 0 | Time between incision and wound closure | 84 | n/a | cumulative incidence | 171.65 mins (49.88) | p value <0.001 | n/a | n/a | unadjusted | no | yes | yes | no | no | Serious |
| Roof | USA | 2016 to 2019 | 2021 | >=19 | 38 | Time between incision and wound closure | 85 | n/a | cumulative incidence | 131.12 mins (33.78) | |  |  |  |  |  |  |  |  |  |

## Surgeon volume and emergency readmissions

| **First author** | **Country** | **Time period** | **Publication year** | **Annual Volume categories** | **Volume midpoint** | **Follow up** | **Patients at risk** | **Numbers with event** | **Type** | **OR/HR** | **Ratio** | **L 95%CI** | **U 95%CI** | **Adjusted or unadjusted** | **Age, Gender, Comorbidity** | **Indication for revision** | **Component Revised** | **Previous Revision Knee Arthroplasty** | **Hospital volume** | **Overall Risk of Bias** |
| --- | --- | --- | --- | --- | --- | --- | --- | --- | --- | --- | --- | --- | --- | --- | --- | --- | --- | --- | --- | --- |
| Roof | USA | 2016 to 2019 | 2021 | <19 | 0 | all cause 90 day readmission | 84 | 10 | cumulative incidence | OR | 1 | 1 | 1 | unadjusted | no | yes | yes | no | no | Serious |
| Roof | USA | 2016 to 2019 | 2021 | >=19 | 38 | all cause 90 day readmission | 84 | 5 | cumulative incidence | OR | 0.468 | 0.14 | 1.38 |  |  |  |  |  |  |  |
